# Multicenter Study Suggests Unsupervised Learning Derived From MRI Identifies Prognostic Subgroups in Prostate Cancer Patients After Prostatectomy

**DOI:** 10.1155/rrp/6424056

**Published:** 2026-06-10

**Authors:** Guoqing Hu, Xiaofeng Liu, Zhangzhe Chen, Bingni Zhou, Hualei Gan, Ruchuan Chen, Liangping Zhou, Yajia Gu, Xiaohang Liu

**Affiliations:** ^1^ Department of Radiology, Fudan University Shanghai Cancer Center, Shanghai, China, shca.org.cn; ^2^ Department of Oncology, Shanghai Medical College of Fudan University, Shanghai, China, fudan.edu.cn; ^3^ Department of Radiology, Fujian Provincial Hospital, Shengli Clinical Medical College of Fujian Medical University, Fuzhou University Affiliated Provincial Hospital, Fuzhou, Fujian, 350001, China, fjmu.edu.cn; ^4^ Department of Pathology, Fudan University Shanghai Cancer Center, Shanghai, China, shca.org.cn

**Keywords:** progression-free survival, prostate cancer, prostatectomy, radiomics, unsupervised learning

## Abstract

**Objective:**

To identify subgroups of patients with prostate cancer (PCa) after radical prostatectomy (RP) based on clinical and magnetic resonance imaging (MRI) radiomics features and evaluate the prognostic value in predicting 5‐year progression‐free survival (PFS).

**Materials:**

Preoperative MRI and clinical data from 400 patients (185 with recurrence) were collected from three centers (one training and two external validation groups). Radiomics features were extracted from index lesions. PFS‐associated clinical and radiomics features were selected by least absolute shrinkage and selection operator (LASSO)‐Cox analysis. The K‐means clustering method was used to identify subgroups and construct a Radiomic‐Clinical model. PFS differences across subgroups were assessed using Kaplan–Meier survival analyses. The predictive performance of the Radiomic‐Clinical model was compared with the European Association of Urology (EAU), University of California, San Francisco (UCSF) Cancer of the Prostate Risk Assessment (CAPRA), and PIPEN models using the concordance index (C‐index).

**Results:**

A total of 5 clinical and 13 radiomics features were selected, and three distinct prognostic subgroups were identified within the Radiomic‐Clinical model. The Radiomic‐Clinical model demonstrated superior predictive accuracy with C‐indices of 0.82 (training group), 0.78 (validation group 1), and 0.79 (validation group 2), outperforming the EAU (0.68, 0.70, and 0.65), CAPRA (0.71, 0.67, and 0.70), and PIPEN models (0.71, 0.70, and 0.68) (*p* < 0.05).

**Conclusion:**

Unsupervised learning using radiomics and clinical data effectively identifies distinct prognostic subgroups in PCa patients after RP, offering superior predictive performance over existing models for 5‐year PFS.

## 1. Background

Prostate cancer (PCa) is the second most common cancer in men, and 20%–30% of the patients experience recurrence within 5 years after radical prostatectomy (RP) [1–3]. Identifying a noninvasive and accurate method to predict the risk of progression in PCa patients after RP is crucial for early development of adjuvant therapy as well as for improving biochemical control and delaying cancer progression. Currently, traditional methods based on clinical data, including the Memorial Sloan Kettering Cancer Center (MSKCC) nomogram [4], the European Association of Urology (EAU) guidelines [5], the University of California San Francisco (UCSF) Cancer of the Prostate Risk Assessment (CAPRA) score [6], and the National Comprehensive Cancer Network (NCCN) guidelines [7] have been applied to assess the survival prognosis of PCa patients. However, clinical and biological heterogeneity are observed among patients and tumors, and predictive effects vary widely between cohorts [8], making accurate risk stratification and prognostic assessment difficult. Magnetic resonance imaging (MRI) has been applied in risk stratification of the prostate, and previous studies [9, 10] have proven that conventional MRI characterization and clinical features are highly efficient in predicting recurrence after RP. In a recent study, a PIPEN model consisting of clinical (International Society of Urological Pathology (ISUP) grading, N stage, etc.) and MRI features (Prostate Imaging Reporting and Data System (PI‐RADS), etc.) yielded a C‐index of 0.74 for the 5‐year progression‐free survival (PFS) of PCa patients undergoing RP, which is better than that of clinical prediction tools (EAU, MSKCC, CRPRA, and Partin risk models) [10]. However, conventional MRI analysis relies mainly on anatomical changes, qualitative reporting paradigms, and subjective interpretations by radiologists, resulting in increased inter‐reader variability and reduced reliability [11].

Radiomics can quantitatively reveal microscopic and macroscopic patterns of cancer lesions in medical images [12]. Some studies have used radiomics features based on MRI images to predict PCa prognosis after RP [13–17], and this approach has been proven to have acceptable predictive efficacy [14]. However, these studies were generally limited by small sample sizes and short follow‐up times, and none of these compared their methods with conventional clinical score. Additionally, most of these studies did not use external validation sets [14, 15], and some only outlined lesions on T2‐weighted imaging (T2WI) [13, 15]. Moreover, all of the studies were limited to clinically local cancer without lymph node metastases, while RP has been increasingly applied in patients with regional PCa (any T, N1, and M0) lymph node metastases according to latest NCCN guidelines [7]. Therefore, the real efficiency of radiomics methods for predicting 5‐year survival in clinical practice is controversial. Moreover, the supervised learning approach used in the above studies was prone to overfitting and poor reproducibility in the training group. In contrast, unsupervised machine learning allows researchers to gain insights into the underlying data distribution, identify novel biomarkers, and understand the inherent structures and relationships within medical data [4]. Previous studies have shown that unsupervised learning can effectively predict recurrence in patients with breast and lung cancer [18, 19].

Therefore, the objective of this study was to identify different prognostic subgroups of PCa patients undergoing RP by utilizing unsupervised learning of clinical factors and biparametric (bpMRI) radiomics features. bpMRI was selected over mpMRI because bpMRI does not require contrast medium and offers comparable efficiency to mpMRI [20, 21], thus is recommended as the primary method for PCa diagnosis by the PI‐RADS guidelines [22] and more readily available in clinical settings. Additionally, the study compared its efficacy in predicting 5‐year PFS to that of the traditional clinical system score.

## 2. Materials and Methods

### 2.1. Subjects

**FIGURE 1:**
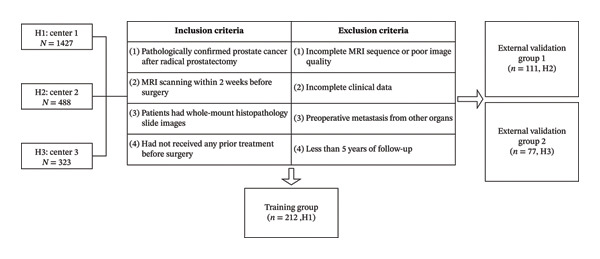
The selection flowchart detailing the criteria for inclusion and exclusion.

This retrospective study was approved by the institutional review boards of the participating centers, and the requirement for informed patient consent was waived. A total of 2238 PCa patients treated at three hospitals between January 2010 and July 2019 were retrospectively enrolled. According to the inclusion and exclusion criteria (Figure 1), a total of 212 eligible patients from Center 1 were assigned to the training cohort, 111 eligible patients from Center 2 to external validation Group 1, and 77 eligible patients from Center 3 to external validation group 2 (Table 1). PFS was calculated as the time from the day of surgery to the appearance of biochemical recurrence (BCR) or the detection of recurrent metastasis by imaging at follow‐up within 5 years after RP. BCR was defined as two consecutive prostate‐specific antigen (PSA) measurements > 0.2 ng/mL in men undergoing RP [5]. Follow‐up for up to 5 years without PFS progression was recorded as 60 months. Follow‐up was conducted in accordance with the EAU guidelines [3] and ended in July 2024. The median follow‐up period was 60 months (IQR: 24–60 months). A total of 185 patients (46%) progressed within five years. All patients underwent radical prostatectomy and received postoperative management in accordance with the NCCN clinical practice guidelines for PCa [7].

**TABLE 1:** Characteristics of all the patients in training and validation groups.

Characteristic		Training (*N* = 212)>	Validation 1 (*N* = 111)	Validation 2 (*N* = 77)
Recurrence		94 (44%)	56 (50%)	35 (45%)

PFS (year)^∗^		60 (24, 60)	59 (26, 60)	60 (24, 60)

Age (year)^∗^		66 (61, 70)	68 (62, 74)	65 (61, 71)

PSA (ng/mL)^∗^		12 (7, 24)	14 (10, 30)	18 (11, 37)

Prostate volume (mL)^∗^		36 (25, 50)	31 (24, 41)	35 (28, 46)

PSAD (ng/mL^2^)^∗^		0.35 (0.20, 0.66)	0.49 (0.30, 1.01)	0.52 (0.28,0.99)

cT stage	1	29 (14%)	25 (23%)	19 (25%)
	2	87 (41%)	36 (32%)	22 (29%)
	3	96 (45%)	50 (45%)	36 (47%)

ISUP	1	21 (9.9%)	6 (5.4%)	7 (9.1%)
	2	70 (33%)	34 (31%)	19 (25%)
	3	65 (31%)	30 (27%)	24 (31%)
	4	20 (9.4%)	17 (15%)	9 (12%)
	5	36 (17%)	24 (22%)	18 (23%)

PI‐RADS	2	6 (2.8%)	2 (1.8%)	4 (5.2%)
	3	27 (13%)	32 (29%)	19 (25%)
	4	64 (30%)	20 (18%)	21 (27%)
	5	115 (54%)	57 (51%)	33 (43%)

EPE score	0	121 (57%)	66 (59%)	40 (52%)
	1	41 (19%)	11 (9.9%)	9 (12%)
	2	16 (7.5%)	18 (16%)	9 (12%)
	3	34 (16%)	16 (14%)	19 (25%)

pT stage	1	29 (14%)	24 (22%)	33 (43%)
	2	75 (35%)	36 (32%)	25 (32%)
	3	108 (51%)	51 (46%)	19 (25%)

Positive surgical margin		31 (15%)	32 (29%)	26 (34%)

Seminal vesicle invasion		34 (16%)	21 (19%)	12 (16%)

Lymph node metastasis		9 (4.2%)	11 (9.9%)	10 (13%)

*Note:* EPE = extraprostatic extension.

Abbreviations: cT stage = clinical T stage, ISUP = International Society of Urological Pathology, PFS = progression‐free survival, PSA = prostate‐specific antigen, PSAD = prostate‐specific antigen density, PI‐RADS = Prostate Imaging Reporting and Data System, pT stage = Pathological T stage.

^∗^Data are medians for continuous variables; data in parentheses are Q1 and Q3.

### 2.2. MRI Protocol

All MRI examinations were performed and interpreted following the Prostate Imaging Reporting and Data System (PI‐RADS) Version 2 guidelines [22] to ensure standardized image acquisition and reporting. All patients underwent bpMRI scans equipped with an 16‐channel body array coil before RP, including conventional axial T2WI and diffusion‐weighted imaging (DWI), and apparent diffusion coefficient (ADC) maps with b values of 0 or 50 and 1500 s/mm^2^ were generated (Table 2).

**TABLE 2:** Magnetic resonance imaging parameters.

Parameter	Scanner 1 (training)	Scanner 2 (training)	Scanner 3 (validation 1)	Scanner 4 (validation 2)
Manufacturer	Siemens Healthcare, Erlangen, Germany	GE Healthcare, Milwaukee, USA	Siemens Healthcare, Erlangen, Germany	Siemens Healthcare, Erlangen, Germany
Model	3T Magnetom Skyra	3T SIGNA Pioneer	3T Magnetom Skyra	3T Magnetom Skyra
T2‐weighted imaging				
Sequence	TSE	FSE	TSE	TSE
TR/TE (ms)	7120/89	3286/85	9040/89	7120/89
Number of signal averages	2	3	3	2
FOV (mm^2^)	220 × 220	220 × 220	200 × 200	220 × 220
Slice thickness (mm)	3.5	4	3.5	3.5
Interslice gap (mm)	0.5	0.5	0	0.5
Matrix	320 × 320	288 × 256	320 × 256	320 × 320
In‐plane resolution (mm^2^)	0.69 × 0.69	0.76 × 0.86	0.63 × 0.78	0.69 × 0.69
Diffusion‐weighted imaging				
Sequence	RESOLVE	FOCUS	RESOLVE	RESOLVE
TR/TE (ms)	4670/63	4581/74.1	6680/66	6500/63
Number of signal averages	2	8	2	2, 2, 6, 6, 6
FOV (mm^2^)	220 × 220	220 × 220	200 × 200	196 × 228
Slice thickness (mm)	3.5	4	3	3
Interslice gap (mm)	1	1	0	1
Matrix	88 × 116	102 × 50	116 × 116	98 × 114
In‐plane resolution (mm^2^)	2.50 × 1.90	2.16 × 4.40	1.72 × 1.72	2.00 × 2.00
*b*‐values (s/mm^2^)	0, 50, 1000, 1500	0, 1000, 1500	50, 1000, 1500	50, 500, 1000, 1500, 2000
Number of *b*‐values	4	3	3	5

*Note:* RESOLVE = readout segmentation of long variable echo trains, FOCUS = field of view optimized and constrained undistorted single‐shot, TR = repetition time, TE = echo time.

Abbreviations: DWI = diffusion‐weighted imaging, FOV = field of view, FSE = fast spin echo, T2WI = T2‐weighted imaging, TSE = turbo spin echo.

### 2.3. Tumor Segmentation

The bpMRI images of all the patients were exported in DICOM format with a postprocessing workstation. The 3D segmentation of region of interest (ROI) was outlined with ITK‐SNAP software (https://www.itksnap.org) on the each conventional axial.

T2WI, DWI (*b* = 1500 s/mm^2^), and ADC images by a radiologist with 8 years of experience in prostate MRI diagnosis who worked under a pathologist with 14 years of experience, and the ROI was reviewed by another imaging physician with more than 15 years of experience in prostate MRI diagnosis. The two radiologists were unaware of the patients’ clinical prognostic information during the outlining process, and any disagreements were resolved by discussion between the two radiologists. The ROI was determined to be either the lesion with the highest Gleason score or the largest lesion among multiple lesions with the highest Gleason score in whole‐mount histopathology slide images [23].

### 2.4. Radiomics Feature Extraction

FeAture Explorer (FAE) software [24] (https://sourceforge.net/projects/feature-explorer/) was initially employed to standardize the preprocessing of both the original and 3D lesion images. This process primarily involved normalizing the gray levels of the images to a scale from zero to one, resampling, and discretizing the grayscale into 25 levels. Subsequently, PyRadiomics, which aligns with the image standardization initiative, was utilized for the extraction of radiomic features. The 2982 features include 54 first‐order texture features, 42 morphological features, 72 gray‐level co‐occurrence matrices (GLCMs), 48 gray‐level run‐length matrices (GLRLMs), 51 gray‐level size zone matrices (GLSZMs), 42 gray‐level dependence matrices (GLDMs), and 264 Laplace and 2112 wavelet variation transformation features. ICC analysis was used to assess observer concordance. The bpMRI images of 30 patients were randomly selected from all patients, and lesions were reoutlined to assess intragroup concordance by an imaging physician with 8–10 years of diagnostic experience in prostate MRI after a period of two months. These 30 lesions were similarly outlined for assessment by another imaging physician with more than 15 years of experience in prostate MRI diagnosis. To address multicenter data heterogeneity, radiomic features extracted from the three centers were harmonized using the ComBat method [25] to adjust for center‐related batch effects. Feature stability across centers was assessed using the ICC; features with ICC < 0.8 were excluded from subsequent analysis to ensure robustness.

### 2.5. Unsupervised Machine Learning and Model Construction

**FIGURE 2:**
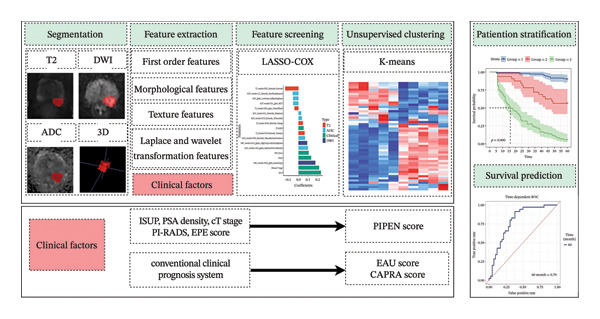
The comprehensive process for the development of clinical and radiomics models, alongside the stratification of risk.

Least absolute shrinkage and selection operator (LASSO)‐Cox analysis was performed on the clinical factors and remaining radiomics features to reduce the dimensionality of the features and noise in the data and select the features related to PFS from the training group. The selected features were subjected to K‐means clustering to identify prognostic subgroups that captured the similarity of feature profiles between subjects. The optimal number of clusters was in the range of 1–20, and the optimal inflection point was determined according to the “elbow rule.” K‐means clustering was initialized randomly using 10,000 iterations to ensure the robustness of the clustering. The final Radiomic‐Clinical clusters identified corresponded to the imaging subtypes of PCa. The entire feature extraction and clustering process is shown in Figure 2. Radiomic‐Clinical models were developed based on the Radiomic‐Clinical clusters. The selected features and optimal number of clusters were then applied to the validation groups.

A simplified five‐item multivariable Cox regression model, termed the PIPEN model, was developed based on a previous study [10]. The model incorporates the following variables: ISUP grade, prostate‐specific antigen density (PSAD), clinical T stage (cT stage), PI‐RADS, and extraprostatic extension (EPE) score. PCa patients were also identified in different prognostic subgroups according to the EAU risk score system and the CAPRA risk score system. Conventional clinical models were developed based on the EAU risk score and the CAPRA risk score.

### 2.6. Statistical Analysis

The statistical analysis was performed using the R software environment, and statistical significance was set to *p* < 0.05. The Kruskal–Wallis test was used for measurement variables, and the chi‐squared test, Kruskal–Wallis rank sum test, Fisher’s exact test, and chi‐square test among clusters were used for categorical variables. Kaplan–Meier survival analyses were used for PFS. The log‐rank test was used to compare PFS among subgroups. The predictive performance of the model was evaluated and compared with the C‐index, utilizing the Somer’ D method [26] to evaluate and compare the *p* values associated with differences in predictive ability across various models.

## 3. Results

### 3.1. PFS Analyses of Conventional Clinical Risk System Subgroups

Patients were categorized into low‐ and intermediate‐risk groups, localized high‐risk groups, and locally advanced high‐risk groups based on the EAU risk score system. They were also categorized into low‐, intermediate‐, and high‐risk groups according to the CAPRA risk assessment system (Table 3). The difference in PFS among the different risk groups in both the training and validation groups was statistically significant (Figures 3(a) and 3(b), all *p* < 0.05). They were also categorized into very low‐, low‐, intermediate‐, high‐, and very high‐risk groups according to PIPEN model. The difference in PFS among the different risk groups in both the training and validation groups was statistically significant (Figure 3(c), all *p* < 0.05).

**TABLE 3:** Clinical risk system and PIPEN score of the patients in training and validation groups.

Characteristic		Training (*N* = 212)	Validation 1 (*N* = 111)	Validation 2 (*N* = 77)	*p*
EAU score	1	27 (13%)	6 (5%)	3 (4%)	0.028
	2	46 (22%)	34 (31%)	18 (23%)	
	3	49 (23%)	23 (21%)	27 (35%)	
	4	90 (42%)	48 (43%)	29 (38%)	

MSKCC score^∗^		37 (17, 67)	34 (12, 58)	30 (13, 60)	0.093

CAPRA score	1	57 (27%)	13 (11%)	10 (13%)	0.009
	2	57 (27%)	34 (31%)	23 (30%)	
	3	98 (46%)	64 (58%)	44 (57%)	

PIPEN score	1	59 (28%)	14 (13%)	13 (16%)	0.004
	2	55 (26%)	28 (25%)	22 (29%)	
	3	22 (10%)	24 (22%)	12 (16%)	
	4	38 (18%)	14 (12%)	8 (10%)	
	5	38 (18%)	31 (28%)	22 (29%)	

*Note:* CAPRA = Cancer of the Prostate Risk Assessment. PIPEN refers to prostate‐specific antigen density, clinical T stage, International Society of Urological Pathology grade group, Prostate Imaging Reporting and Data System category, extraprostatic extension score.

Abbreviations: EAU = European Association of Urology, MSKCC = Memorial Sloan Kettering Cancer Center.

^∗^Data are medians for continuous variables; data in parentheses are Q1 and Q3.

FIGURE 3Kaplan‐Meier survival curves of EAU score (a), CAPRA score (b), PIPEN score (c), and Radiomic‐Clinical clustering (d) in the training and external validation groups. EAU, European Association of Urology; CAPRA, Cancer of the Prostate Risk Assessment. PIPEN refers to prostate‐specific antigen density, clinical T stage, International Society of Urological Pathology grade group, prostate imaging reporting and data system category, extraprostatic extension score.

**Figure:**
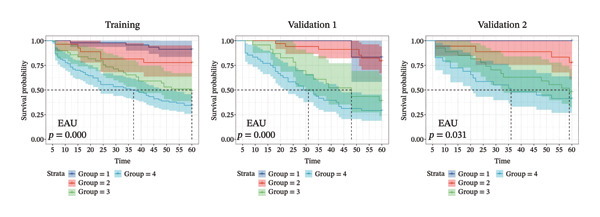
(a)

**Figure:**
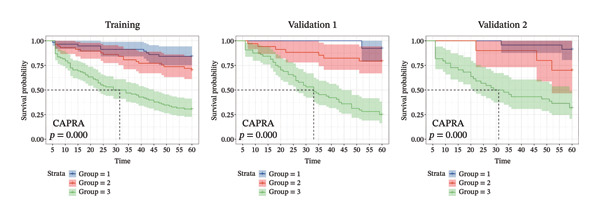
(b)

**Figure:**
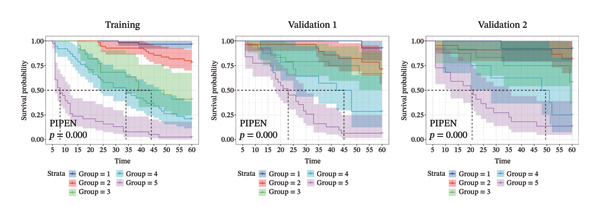
(c)

**Figure:**
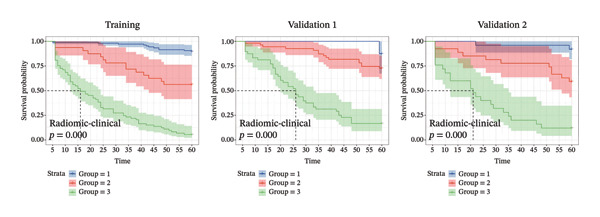
(d)

### 3.2. PFS Analyses of Unsupervised Clustering Subgroups

**FIGURE 4:**
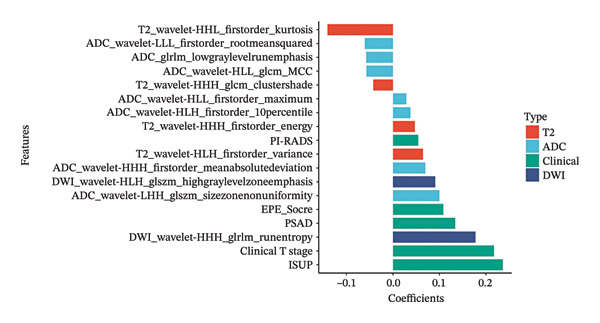
LASSO‐Cox analysis model on CT images, 5 clinical and 13 radiomics features were selected.

In the training group, 5 clinical factors and 13 radiomics features were retained after LASSO‐Cox analysis (Figure 4). These features were clustered with the K‐means method, and the three risk subgroups were identified as the optimal number of Radiomic‐Clinical clusters. In the training group, the 5‐year PFS rates were significantly different among the patients from three subgroup (90%, 56%, and 5%, Figure 3(d); *p* < 0.05), and those subgroups were then identified as the low‐, intermediate‐, and high‐risk subgroups according to the length of median PFS, respectively. The 5‐year PFS rates were significantly different among the patients from three subgroup in external validation group 1 (90%, 73%, and 17%, Figure 3(d); *p* < 0.05) and external validation group 2 (92%, 59%, and 12%, Figure 3(d); *p* < 0.05). The redistribution of the study patients according to unsupervised Radiomic‐Clinical clustering is shown in Table 4. Expect for PIPEN score, overall associations among the EAU and CAPRA score were observed (*p* < 0.001).

**TABLE 4:** The redistribution of the study patients according to unsupervised Radiomic‐Clinical clustering.

**Characteristic**		**Cluster 1**	**Cluster 2**	**Cluster 3**	***p***
**Training**		**(*N* = 107)**	**(*N* = 32)**	**(*N* = 73)**	

EAU score	1	21 (20%)	3 (9%)	3 (4%)	< 0.001
	2	39 (36%)	5 (16%)	2 (3%)	
	3	19 (18%)	13 (41%)	17 (23%)	
	4	28 (26%)	11 (34%)	51 (70%)	

CAPRA score	1	49 (46%)	6 (19%)	2 (3%)	< 0.001
	2	34 (32%)	8 (25%)	15 (21%)	
	3	24 (22%)	18 (56%)	56 (77%)	

PIPEN score	1	28 (26%)	10 (31%)	21 (29%)	0.740
	2	25 (23%)	11 (34%)	19 (26%)	
	3	14 (13%)	1 (3%)	7 (10%)	
	4	19 (18%)	4 (13%)	15 (20%)	
	5	21 (20%)	6 (19%)	11 (15%)	

**Validation 1**		**(*N* = 8)**	**(*N* = 55)**	**(*N* = 48)**	

EAU score	1	0 (0%)	5 (9%)	1 (2%)	< 0.001
	2	2 (25%)	28 (51%)	4 (8%)	
	3	3 (38%)	7 (13%)	13 (27%)	
	4	3 (38%)	15 (27%)	30 (62%)	

CAPRA score	1	2 (25%)	10 (18%)	1 (2%)	< 0.001
	2	5 (62%)	22 (40%)	7 (15%)	
	3	1 (12%)	23 (42%)	40 (83%)	

PIPEN score	1	1 (12.5%)	9 (16%)	4 (8%)	0.899
	2	2 (25%)	13 (24%)	13 (27%)	
	3	1 (12.5%)	12 (22%)	11 (23%)	
	4	2 (25%)	5 (9%)	7 (15%)	
	5	2 (25%)	16 (29%)	13 (27%)	

**Validation 2**		**(*N* = 25)**	**(*N* = 27)**	**(*N* = 25)**	

EAU score	1	2 (8.0%)	1 (3.7%)	0 (0%)	0.001
	2	10 (40%)	8 (30%)	0 (0%)	
	3	6 (24%)	11 (41%)	10 (40%)	
	4	7 (28%)	7 (26%)	15 (60%)	

CAPRA score	1	7 (28%)	2 (7%)	1 (4%)	< 0.001
	2	11 (44%)	10 (37%)	2 (8%)	
	3	7 (28%)	15 (56%)	22 (88%)	

PIPEN score	1	4 (16%)	6 (22%)	3 (12%)	0.857
	2	6 (24%)	7 (26%)	9 (36%)	
	3	6 (24%)	3 (11%)	3 (12%)	
	4	3 (12%)	2 (7%)	3 (12%)	
	5	6 (24%)	9 (33%)	7 (28%)	

*Note:* PIPEN refers to prostate‐specific antigen density, clinical T stage, International Society of Urological Pathology grade group, Prostate Imaging Reporting and Data System category, extraprostatic extension score. CAPRA = Cancer of the Prostate Risk Assessment.

Abbreviation: EAU = European Association of Urology.

### 3.3. Predictive Efficacy of the Model

In predicting the 5‐year PFS of PCa patients, conventional EAU, CAPRA, and PIPEN models showed closed C‐indices of 0.68, 0.71, and 0.71 in the training group (Table 5, *p* > 0.05). The Radiomic‐Clinical model had better predictive ability than all the above models (all *p* < 0.05), with C‐index of 0.82. The C‐indices of EAU, CAPRA, and PIPEN models still show no significant difference among each other in external validation groups 1 (0.70, 0.67, and 0.70) and 2 (0.65, 0.70, and 0.68) (all *p* > 0.05). The C‐index of Radiomic‐Clinical model in validation groups 1 (0.78) and 2 (0.79) were also significantly higher than the above modes (all *p* < 0.05).

**TABLE 5:** Predictive efficacy of clinical models and unsupervised learning model.

Model	Training	Validation 1	Validation 2
EAU	0.68	0.70	0.65
CAPRA	0.71	0.67	0.70
Modified PIPEN	0.71	0.72	0.68
Radiomic‐Clinical	0.82	0.76	0.79

*Note:* CAPRA = Cancer of the Prostate Risk Assessment. PIPEN refers to prostate‐specific antigen density, clinical T stage, International Society of Urological Pathology grade group, Prostate Imaging Reporting and Data System category, extraprostatic extension score.

Abbreviation: EAU = European Association of Urology.

## 4. Discussion

In this study, we used clinical factors and bpMRI radiomics features to predict the 5‐year PFS of patients with PCa from three centers, stratified the patients according to risk with unsupervised clustering methods, and achieved better performance than existing clinical prognostic systems. These findings suggest that the unsupervised clustering‐based method can effectively identify patients at high risk of postoperative recurrence among patients with PCa.

In terms of clinical factors, this study revealed that higher PSAD, cT stage, PI‐RADS score, EPE score, and ISUP were associated with a greater risk of recurrence. It is interesting that features referring to conventional T2WI (PI‐RADS and EPE‐score) still retain substantial clinical value in the prognosis prediction of disease. A previous study by Luzzago et al. [10] demonstrated that a PIPEN model based on traditional imaging features can help predict the risk of postoperative recurrence of PCa, which is similar to our result. Another study on breast cancer also suggests that peritumoral edema on T2WI is associated with poor prognosis in mass‐type cancer, whereas its reduction after neoadjuvant chemotherapy indicates a more favorable outcome [27]. Those result indicated that even in clinical settings, besides the diagnosis of current prostate disease, the assessment on conventional images by radiologists could also provide preliminary clues for adverse prognosis at further stage, and bring about further and quantitative exam and evaluation.

However, the PIPEN model in a previous study [10] did not show better predictive efficacy in our centers. This difference possibly occurred because there were significantly more PCa patients at intermediate and advanced stages in this study than in Europe and the United States (46% at T3), and thus, the original PIPEN model might not be suitable for conventional clinical prediction systems, which is consistent with a recent study [28]. Jia [14] and Marturano et al. [29] reported that PCa predictive performance was further improved when clinical data were complemented with radiological features, which indicated that even with radiology and radiomics data, these clinical factors are still essential for prognosis assessment. However, seminal vesicle invasion (SVI) was not included, which was inconsistent with the results of previous studies [10, 30, 31]. This may be because the quantitative T2WI and DWI signal and ADC values have high predictive value for SVI and make SVI insignificant in LASSO‐Cox regression [32, 33].

Radiomics features based on the bpMRI were significantly associated with prognosis. The radiomics features with the higher coefficient, such as T2_wavelet‐HHL‐firstorder‐Kurtosis, DWI_wavelet‐HHH_glrlm_runentropy, and ADC_wavelet‐LHH_glszm_SizeZoneNonUniformity, focuses on heterogeneity‐associated features, which is consistent with previous studies that higher heterogeneity always indicates higher aggressivity [34]. Some studies further suggest that heterogeneity within tumors and metastatic sites, even within the same patient, is believed to be a major cause of treatment failure [35, 36]. Interestingly, some features, such as T2_wavelet‐HHL_firstorder_Kurtosis, DWI_wavelet‐HHH_glrlm_RunEntropy, ADC_wavelet‐HHH_firstorder_MeanAbsoluteDeviation, and ADC_wavelet‐HLL_firstorder_Maximum, also acted in several previous studies [13–15]. Although the coefficients were lower than those of the clinical data and the mechanism of these relationships is still unclear, this finding indicates that predicting the prognosis by radiomics has stable reproducibility for further application.

In our study, compared with the PIPEN and conventional clinical models, the Radiomic‐Clinical model showed greater predictive efficacy, with the C‐indices of 0.82, 0.78, and 0.79 in the training group, external validation group 1, and external validation group 2, respectively. Radiomics could extract and analyze as many meaningful deep quantitative features from images as possible for decision support [37, 38] and provide information that was not detected by the overall image and clinical data. Several studies [39] have proven the relationship among radiomics, immunochemical formation, and gene mutation. Therefore, we infer that the Radiomic‐Clinical model could provide more intensive assessment on the prognosis due to its combination of clinical data and relevant information from radiomics features.

There were also some studies using machine learning to predict PCa recurrence after RP. A study [13] developed a random forest radiomic model using T2WI from 225 patients to predict PCa recurrence within two years postsurgery, achieving an AUC of 0.78. Another study [17] of 198 bpMRI patients developed RadClip, a radiomic‐clinicopathologic nomogram, which predicted BCR‐free survival postsurgery with a C‐index of 0.77, surpassing CAPRA’s 0.68 and Decipher’s 0.51. Nevertheless, the methodologies employed in the aforementioned studies predominantly rely on supervised learning techniques, which are susceptible to overfitting and may result in limited reproducibility within the training dataset. Compared with these articles that focused on two‐end studies and on the probability of recurrence after RP, our study used the K‐means method for prognostic risk stratification of PCa patients. The results also showed a good level of prediction, with cluster subgroup 3 having the worst 5‐year PFS prognosis and cluster subgroup 1 having the best prognosis, both in the training and external validation groups (*p* < 0.05). This finding suggests that we can identify three prognostic subgroups of PCa shortly after RP and provide specific treatment. For example, active neoadjuvant treatments such as ADT and radiotherapy should be applied to Group 3 (high risk subgroup) patients, closed follow‐up could be carried out in Group 1 (low risk subgroup), and moderate attention should be given to Group 2 (intermediate‐risk subgroup). Moreover, compared with the previous study [13–17], we included patients with regional lymph node metastases that were confirmed to benefit from RP according to the latest NCCN guidelines and discovered a higher efficiency than conventional clinical score, which further confirmed the value of the Radiomic‐Clinical model in prognosis assessment.

This study has several limitations. First, this study was retrospective, which may be subject to selection bias. Second, this study did not outline the enhanced images, which may have lost some useful features. In addition, this study used manual ROI sketching, which is time‐consuming and laborious, and in the future, we aim to find a method that can automatically outline the ROI. Third, this study was validated in relatively small external validation sets, and future validation will be needed to expand the sample size for further validation. Fourth, while our radiomic analysis incorporated DWI with a maximum *b*‐value of 1500 s/mm^2^—which falls within the high‐*b*‐value range (> 1000 s/mm^2^)—recent studies have demonstrated that diffusion data acquired at higher *b*‐values (e.g., 2000 s/mm^2^) can provide additional microstructural information that may further enhance the characterization of PCa [40–42]. Specifically, these studies have shown improved differentiation between benign tissue, low‐grade PCa, and high‐grade PCa using *b*‐values of 2000 s/mm^2^. Therefore, incorporating higher‐*b-*value DWI features represents a promising direction for future work to further refine prognostic stratification.

## 5. Conclusion

In summary, in this study, we found that unsupervised learning–based bpMRI radiomics features and clinical factors have high predictive prognostic value, and these features can help identify high‐risk patients at an early stage, adjust the treatment regimen, and improve the prognosis of patients.

## Author Contributions

Conception and design: Guoqing Hu, Xiaofeng Liu, and Xiaohang Liu. Administrative support: Xiaohang Liu and Yajia Gu. Provision of study materials or patients: Xiaofeng Liu, Bingni Zhou, and Ruchuan Chen. Collection and assembly of data: Guoqing Hu, Xiaofeng Liu, and Zhangzhe Chen. Data analysis and interpretation: Guoqing Hu, Xiaohang Liu, and Yajia Gu. Manuscript writing: all authors.

## Funding

This work was supported by the Climbing foundation of National Cancer Center of China (no. NCC201909B03).

## Disclosure

This manuscript has been previously deposited as a preprint on Research Square (https://www.researchsquare.com/article/rs-5864691/v1).

## Ethics Statement

This retrospective study was approved by the institutional review boards of the participating centers, and the requirement for informed patient consent was waived.

## Conflicts of Interest

The authors declare no conflicts of interest.

## Data Availability

The datasets used and analyzed during the current study are available from the corresponding author upon reasonable request.
